# Are There Differences in the Health Outcomes of Mothers in Europe and East-Asia? A Cross-Cultural Health Survey

**DOI:** 10.1155/2014/856543

**Published:** 2014-11-27

**Authors:** Eva Mautner, Chie Ashida, Elfriede Greimel, Uwe Lang, Christina Kolman, Daniela Alton, Wataru Inoue

**Affiliations:** ^1^Department of Obstetrics and Gynaecology, University Hospital Graz and Medical University of Graz, Auenbruggerplatz 14, 8036 Graz, Austria; ^2^Graduate School of Education, Hiroshima University, 1-1-1 Kagamiyama, Higashihiroshima, Hiroshima 739-8524, Japan

## Abstract

The aim of the current study was to investigate differences in quality of life outcomes and depression of mothers in East-Asia and Central Europe. 170 women in Japan and 226 women in Austria with children between 3 and 5 answered the same cross-culturally validated questionnaires. The Quality of Life Questionnaire from the WHO (WHOQOL-Bref), the Patient Health Questionnaire (PHQ-2), the Sense of Coherence Scale (SOC-13), a Social Support Scale (MSPSS), and questions on gender orientation were used. In all dimensions of QOL (physical, psychological, social, and environmental) Japanese women had lower QOL scores compared to Austrian mothers (*P* < 001). Seven percent of women in both countries experienced major depression. In both countries sense of coherence, experienced stress level, satisfaction with income, social support, and gender roles had an influence on QOL and depressive symptoms. Mothers in Japan consider life events less comprehensible, manageable, and meaningful and experience less support. Consequently, creating an environment where fathers could be more involved in child rearing and mothers have more opportunities to choose between life styles and working and social environments would improve QOL not only in Japanese mothers but also in other countries all over the world.

## 1. Introduction

Early motherhood is expected to be a time of happiness for mothers. Instead women cannot always cope with this new situation. It is known that, worldwide, 10 to 20 percent of young mothers experience postnatal depression during the first years [1]. The multiple roles that women fulfill as wives, mothers, caretakers, and employees expose them to greater risk of experiencing mental health disturbances. Among reproductive-age women, depression produces the highest burden of disease in both developed and developing countries [2, 3]. It is widely acknowledged that maternal health heavily impacts children's health. Parents, especially mothers, are the primary gatekeepers of their children's health worldwide [3, 4].

Quality of life (QOL) is a useful concept measuring the health state experienced by women. It has become recognised and established as an outcome variable and health status indicator in medical and public health research. According to the WHO, QOL is an “individual's perception of their position in life in the context of the culture and value systems in which they live and in relation to their goals, expectations, standards and concerns” [5]. The concept of QOL is based on the WHO definition of health that describes “health as a state of complete physical, mental and social well-being and not merely the absence of disease or infirmity.” The main dimensions of QOL are perceived physical health, psychological well-being, social relationships, and environmental factors. In measuring QOL, the WHOQOL Group takes the view that it is important to know how satisfied or bothered people are by important aspects of their life, and this interpretation will be a highly individual matter [6].

In Japan and Austria the birth rate in 2012 was 1.4 children per woman. The reasons may be similar: an extended youth period, increased demands for education from employers, and difficulty in combining work and family life [7]. There are differences in Japan and Austria according to the employment patterns among couple families with children aged 0 to 14. Although dual-earnership is the most common model among couple families in the majority of OECD countries, in Austria, Germany, Switzerland, the UK, and the Netherlands the most usual arrangement is a one-and-a-half earner household. In Japan single income families are more common. According to the OECD in the year 2000 “Social Safety and Security” was high in Japan and Austria because of the low unemployment rate. However, the costs for child rearing are higher in Japan compared with the OECD average and Austria. Furthermore, the possibility of balancing work and home life is more difficult in Japan compared to the OECD average. In traditional Asian cultures, women are expected to play the major role in child rearing [3]. The involvement of fathers in European countries has increased during recent years and it has been shown that it has a benefit on the health and well-being of men, as well as women and children [8]. However, the ratio of male part-time employment and division of roles in housework is low in Austria [9].

An international comparison survey conducted by the Japanese Government reported that the proportion of mothers who considered raising children enjoyable was 44% among Japanese mothers, while the percentage was 81% in the United States [10]. The proportion of Japanese mothers who report feeling stressed during child rearing has increased in the last two decades, from 11% in 1980 to 33% in 2002 among mothers of 18-month-old babies [11]. In previous studies in Austria it was found that social support from the father and significant others, the economic situation, previous high depressive symptoms, and resilience had an influence on QOL and depressive symptoms in early motherhood [12–14]. Many other authors have found perceived social support to be a good predictor of psychological well-being [15].

A growing number of studies have additionally examined sense of coherence (SOC) as a resilience measure. It has been observed that some people, regardless of major stressful situations and severe hardships, stay healthy, while others do not. Consequently the sense of coherence concept was formulated. It is a “global orientation, a pervasive feeling of confidence that the life events one faces are comprehensible, that one has the resources to cope with the demands of these events, and that these demands are meaningful and worthy of engagement” [16]. The construct of SOC has received considerable attention across a range of cultures and research settings [17]. SOC seems to have an impact on QOL, as shown in a systematic review. It enables individuals to effectively mobilize resources to cope with a variety of stressors and promote healthy well-being [18]. In a comparative cross-cultural health survey conducted in three countries in Central Europe it was found that QOL, SOC, and subjective state of health were rated highest in the Austrian sample followed by the Italian and Slovenian samples [19].

The aim of the current study was to investigate differences in QOL outcomes and depression of mothers in Japan and Austria. Additionally, the impact of influential factors such as sense of coherence, the experienced stress level, social and gender roles, the financial situation, and social support was examined. We expected differences in quality of life of mothers in Japan and Austria because of different living conditions in East-Asia and Central Europe.

## 2. Materials and Methods

### 2.1. Study Design

Participants in this questionnaire survey were women with at least one child between the age of 3 and 5 years in Austria and Japan, who had reading and writing abilities in Japanese and German. The survey was conducted in kindergartens in Hiroshima, Japan, and kindergartens in Styria, Austria. Data were collected independently in both countries using the same cross-culturally validated questionnaires from January 2014 to March 2014. Mothers were invited to take part in the study from the study team and got an information letter. The kindergarten supported the study by collecting the questionnaires anonymously in boxes. No financial compensation was offered to the women for their participation. All participants agreed to take part in the study. A different response rate was expected in Japan (80%) and Austria (40%), as studies in Japan and Austria have shown [20, 21]. The Institutional Review Boards from Austria (Graz Medical University) and Japan (Hiroshima University) approved the study.

### 2.2. Questionnaires

#### 2.2.1. Quality of Life

The WHOQOL-BREF, a 26-item short version of the WHOQOL-questionnaire, was used to measure QOL. The WHOQOL-BREF is a cross-culturally valid generic questionnaire which measures four specific domains of QOL, physical, psychological, social, and environmental domains, and a global QOL scale. This instrument was developed simultaneously in more than 15 cultural settings. It can be used to assess variations in QOL across different cultures, to compare subgroups within the same culture, and to measure changes across time in response to changes in life circumstances [22, 23]. Scores range from 0 to 100 with higher scores indicating better quality of life on all domains.

#### 2.2.2. Depressive Symptoms

Two items from the Patient Health Questionnaire, the PHQ-2, were used to measure depressive symptoms in both populations. Scores range from 0 to 6 with higher scores indicating more depressive symptoms. A cut-off point of 2/3 is recommended. A PHQ-2 score > or = 3 had a sensitivity of 0.77 and a specificity of 0.95 for major depression in a Japanese population [24]. In a German sample a cut-off score of 2/3 had a sensitivity of 0.87 and a specificity of 0.78 for major depression [25].

#### 2.2.3. Perceived Stress

Perceived stress was measured with a single scale: women had to estimate the degree of stress they felt during the last three days on a scale from 0% (no stress at all) to 100% (very much stress).

#### 2.2.4. Sense of Coherence (SOC-13)

The 13-item short form of the SOC was used to measure sense of coherence (SOC-13). Subjects were asked to respond to a series of statements using a seven-point scale from 1 to 7. A total score is summed, which can range from 13 (low sense of coherence) to 91 (high sense of coherence). The SOC scale consists of three dimensions: the comprehensibility, the manageability, and the meaningfulness component. The questionnaire has been used on subjects from Western cultures and countries such as Japan, China, Thailand, and South Africa. The mean SOC seems to be independent of the cultural context.

#### 2.2.5. Gender Orientation

Five questions were used to measure gender orientation: one general question about gender roles and one question about the mother's, father's, woman's, and man's role. The scales range from 1 (do not agree) to 5 (agree completely). The questions were developed by Roth (1980) [26] in previous gender studies.

#### 2.2.6. Child Factors

Two questions were asked about attitudes to children concerning difficulties raising a child and meaning of a child. Items are rated on a 7-point Likert-scale ranging from 1 (very strongly disagree) to 7 (very strongly agree).

#### 2.2.7. Social Support

The Multidimensional Scale of Perceived Social Support (MSPSS) was used to measure social support. Twelve items are assessing three sources of support as follows: family, friends, and significant others. Items are rated on a 7-point Likert-scale ranging from 1 (very strongly disagree) to 7 (very strongly agree). The lowest score in subscales is 4, and the highest is 28. The lowest overall scale score is 12, and the highest is 84.

#### 2.2.8. Psychosocial Data Sheet

The psychosocial data sheet included questions of age, education, living situation, number of children, employment status, and questions about financial situation.

### 2.3. Statistics

Data were analysed using the Statistical Package for the Social Sciences (SPSS Version 20.0). Descriptive statistics were used to characterize the study sample in terms of sociodemographic variables. The independent samples *t*-test was conducted to test for differences between Japan and Austria in age, number of children, and financial situation. The chi-square test was used to test for between-group differences in education, living situation, and employment status. The independent sample *t*-test was performed to test for differences between the countries in QOL, depressive symptoms, perceived stress, sense of coherence, gender orientation, importance of a child, difficulty of child rearing, and social support. The level of significance was determined with *P* < 0.05.

In order to evaluate the impact of psychological characteristics on QOL, besides cultural differences, an analysis of multiple stepwise regressions according to the General Linear Model was performed in each country. In this analysis, factors such as sense of coherence, gender orientation, the importance of having a child, difficulties in child rearing, the stress-level, social support, the economic situation, age, and number of children were included. The data in Japan and Austria were analysed to determine if different parameters contributed to the QOL-dimensions and depression.

## 3. Results

### 3.1. Sample Characteristics

The study sample consisted of 170 women from Japan and 227 women from Austria with at least one child in kindergarten, response rate being 85% in Japan and 50% in Austria. The mean age of Austrian participants was 36.31 and in Japanese women 37.32 years. More of the Japanese women had higher education (90%) compared to Austria (58.1%). Austrian mothers had post-compulsory education more often than Japanese mothers (30.8% versus 9.4%, resp.).

There are significantly more single mothers in Austria (10.1%) compared to Japan (1.8%). In Japan mothers live significantly more often with their partner and grandparents compared to Austrian mothers (11.8% versus 6.2%). In the Japanese population the number of children was significantly higher (mean 2.21, SD = 0.72) compared to Austria (mean 2.04, SD = 0.82). In both countries women had between 1 and 6 children. The employment status was significantly higher in Austria with 15% full-time employed and 65.5% part-time employed compared to Japan with a full-time employment rate of 13.5% and a part-time employment rate of 17.1%. 89.4% of Austrian mothers were either very satisfied with making a good living or satisfied, compared with 57.7% of Japanese mothers (Table 1).

### 3.2. Main Results

High significant differences were found between Japanese and Austrian mothers in all QOL dimensions. Japanese mothers had significantly lower scores on the physical (M = 63.36, SD = 14.13 versus M = 80.28, SD = 12.66), psychological (M = 59.14, SD = 13.76 versus M = 73.75, SD = 14.50), social (M = 63.66, SD = 14.170 versus M = 73.0, SD = 19.13), and environmental QOL-dimensions (M = 59.06, SD = 13.18 versus M = 80.35, SD = 13.17) compared to Austrian mothers (*P* < 0.001). However there were no differences in both countries in depressive symptoms. 7.6% of Japanese and 7.0% of Austrian mothers were equal to or above a score of 3 on the PHQ-2, indicating a possible major depression (Table 2).

Japanese and Austrian mothers did not differ in experienced stress level. However women in Austria had significantly better sense of coherence scores. Consequently Austrian mothers experience their life as more comprehensible, manageable, and meaningful compared to Japanese women (Austria: M = 68.67, SD = 12.20; Japan: M = 57.82, SD = 12.17).

Japanese mothers are significantly more traditionally oriented. They think less that women should share their duties at home and at their jobs (general role) and more that men should be the main financial resource for a family (men's role). Japanese women also think that a woman should mainly care for her family and not for her job (women's role). In both countries women consider that a father should spend much time with the children during the week, not only at weekends (father's role). Austrian mothers however think that it is mainly the mother's responsibility to care for their children compared to Japanese mothers (mother's role). In both countries a child is very important. Japanese mothers experience child rearing as significantly more difficult than Austrian mothers. As for social support, mothers from both countries get the same amount of support from the family. In addition, women in Austria get significantly more support from important others, friends, and partners compared with Japanese mothers (see Table 3).

### 3.3. Influences on Depression and Quality of Life

The experienced stress level and sense of coherence had an influence on depression in Austria and Japan. The higher the experienced stress was and the lower the sense of coherence was, the more women expressed depressive symptoms. In Japan additionally less social support from the family explained more depressive symptoms. All results are shown in Table 4.

Factors for better physical QOL were a lower stress level and better sense of coherence in Austria and Japan. In Austria the satisfaction with income was another impact factor for better physical QOL and in Japan social support from the family.

In Austria and Japan explanatory factors for better psychological QOL were higher sense of coherence scores and higher scores on social support from the family. In Austria “higher satisfaction with income” and in Japan a “lower experienced stress level” had an additional impact on better psychological QOL.

Social QOL was explained in both countries by higher sense of coherence scores and more social support from friends and from the partner. Other explanatory factors for better social QOL were in Austria social support from the family and in Japan the mother's and father's role. Concerning the mother's role, this means that Japanese women who do not think that it is predominantly their responsibility to care for the children and the father should spend not much time with their children have better social QOL.

In both countries factors for better environmental QOL were satisfaction with income and better sense of coherence. In Austria other impact factors included social support from friends and women's role. Concerning women's role, this means that the less women think that they should mainly care for their family not for their job, the more they had better environmental QOL. In Japan social support from important others was another impact factor for better environmental QOL (Table 4).

## 4. Discussion 

The primary purpose of the current study was to examine differences in quality of life outcomes and depression of mothers in Japan and Austria since women seem to be the primary gatekeepers of children's health worldwide and maternal health impacts heavily children's health [3, 4]. Participants of the current study were mothers with at least one child between the age of 3 and 5 years in kindergarten. Women in both countries differed according to culturally expected variation in demographic characteristics such as employment status, education, and living situation. In our sample 20% of Austrian women were not employed compared to 70% of Japanese women. This is in line with the OECD report (2010), underlining that the possibility of balancing work and home life in Japan is more difficult than in other OECD countries. The percentages for flexibility of work styles and reasonable work hours are very low in Japan [9]. Living alone with children seems to be more possible in Austria. Ten percent of mothers in the Austrian sample lived alone without a partner. This can be explained by the fact that social tolerance for diversity in Austria seems to be higher and Austrian mothers feel more independent to run their own lives [9]. The education level and number of children in both countries were above the average [27, 28]. One reason might be that well educated mothers were more interested in our study and were more likely willing to contribute to the current QOL surveys. Many women expressed their interest in the outcomes of the current study. In Austria, kindergartens which agreed to take part were mainly in wealthy regions. In Japan kindergartens are attached to the University and well-educated women send their children to these kindergartens. This association with the kindergartens in Japan might have been one reason for the high response rate, which has also been observed in previous studies [20]. In Japan, 90% of mothers had an academic degree. Most of them had a bachelor degree. Austrian women are more likely to have a master degree, because the bachelor system has been established recently. In addition nurses or teachers in kindergarten are not trained at universities in Austria. Consequently the different education systems in both countries also may have caused the dissimilarities in the education level in our study groups. In further cross-cultural studies we recommend additionally to ask about the years of education.

We expected differences in QOL of mothers in Japan and Austria because of different living situations. The results were in line with this hypothesis. In all QOL dimensions, physical, psychological, social, and environmental QOL, Japanese mothers had lower QOL compared to Austrian mothers. However, the large differences were not expected because both countries have high living standards. On the other hand even in Europe Austria seems to have high QOL levels compared, for example, to Slovenia and Italy, as a previous survey in Central Europe has shown [18].

Sense of coherence has been described as a health resource [17]. In the current study we found that Japanese and Austrian mothers differed in SOC. Austrian mothers experience their life as more comprehensible, manageable, and meaningful compared to Japanese mothers. Compared with the OECD report, which indicated a low percentage of Japanese people believing that they are free to run their own lives and that individual human rights are respected, this seems to be explainable, adding the fact that the potential of young people for autonomy is lower in Japan than the OECD average [9].

Further differences were found in gender orientation between Japanese and Austrian mothers, indicating a more traditional role for Japanese mothers. In this respect Japanese women see their duties more at home, and mothers feel more responsible to care for the children and believe a man should be the main financial resource for the family. Nevertheless, in both countries women had highest and similar scores on the father's role. This means that in both countries women believe that a father should spend much time with the children during the week, not only at weekends. Reality in Japan seems to be different. Almost 30% of Japanese employees work more than 50 hours a week, compared to 3% in Austria [27, 28].

To examine which factors have the main impact on the QOL dimensions and depressive symptoms in each country, stepwise regression analyses were conducted. We confirmed other studies, which found that sense of coherence, perceived social support, and income are good predictors for well-being [14, 17]. Even though significant differences between Japanese and Austrian mothers in QOL levels exist, in both countries the main important factor in the current study was SOC, which had an impact on all QOL domains and depression. Other factors in both countries were experienced stress level, social support, especially from the family, income, and gender roles. However the influence patterns on QOL were different in Japan and Austria. In Austria satisfaction with income had a more important influence compared to Japan. This might be related to the real income. Consequently poverty of women seems to be a main risk factor in Austria for lower physical, psychological, and environmental QOL. In Japan satisfaction with income had an impact just on environmental QOL, but women were in general not as satisfied with income compared to Austrian mothers. Stress seems to be a more important factor in Japan. It has an impact on psychological QOL, depression, and physical QOL (in Austria just on depression and physical QOL). Social support of the family seems to be more important in Japan for depression, physical QOL, and psychological QOL. This might be explained by the absence of other social resources in Japan, like social support of friends and important others, which was considered as additional resources in Austria. To summarize, it seems that mothers in Japan have lower QOL and a much less feeling of confidence that the life events they face are comprehensible, that they have the resources to cope with the demands of everyday life, and that they are meaningful and worthy of engagement.

### 4.1. Limitations and Suggestions

The current study is a QOL study in two countries in Central Europe and in East-Asia. Our study was a convenient study with a high academic level. It is difficult to judge, if Austria is representative for Europe and Japan representative for Asia. In Europe and Asia there are huge differences between the countries, so you might not find the typical European or Asian country. Austria is comparable with Germany, Switzerland, the UK, and the Netherlands, where one-and-a-half earner households are equally common [9]. Japan can be considered as a highly developed Asian country [9]. Consequently we may conclude that both countries are comparable. Currently there are not many QOL data available for mothers in Europe or other countries worldwide, so this might be a first step in exploring QOL of mothers.

In Japan it seems that mothers still do not have enough control over their lives and have less support, which could have caused the exceptionally low QOL. In particular in the environmental and physical QOL domains, women in Japan experience subjectively lower QOL. However we did not investigate QOL of fathers in the current study, and this could give an additional insight into the well-being of families in both countries.

## 5. Conclusions

Over the world different environmental conditions for mothers are found, which should be investigated. Evaluating QOL might be an approach to evaluate the health outcomes of mothers and discuss further interventions. There is evidence to suggest that mother's psychosocial and mental health have significant effects on children's growth, nutritional status, and emotional development [4]. Consequently improving the well-being or QOL of mothers should have a high priority all over the world. We would advocate QOL research in mothers globally to find out where needs of improvement can be found. Another step is to develop strategies to improve QOL and well-being of mothers. Creating an environment where fathers could be more involved in child rearing and mothers have more possibilities to choose between life styles and working and social environments could improve QOL not only in Japanese mothers but also in other countries and might improve the QOL of fathers and children.

## Figures and Tables

**Table 1:** Demographic characteristics of Japanese and Austrian mothers with at least one child between the age of 3 and 5 in kindergarten.

	Japan (*N* = 170)		Austria (*N* = 227)	*P*
Age				
M ± SD	37.32 ± 4.05	>	36.31 ± 4.85	0.030
Range	27–50		24–52	
Education				
Compulsory	2 (1.2%)		25 (11.0%)	0.000
Post-compulsory	16 (9.4%)	<	70 (30.8%)	
Higher education/university	152 (89.4%)	>	132 (58.1%)	
Living situation				
With children	3 (1.8%)	<	23 (10.1%)	0.005
With partner and children	144 (84.7%)		186 (81.9%)	
With partner, children, and grandparents	20 (11.8%)	>	14 (6.2%)	
With children and grandparents	3 (1.8%)		3 (1.3%)	
Others			1 (0.4%)	
Number of children				
M ± SD	2.21 ± 0.72	>	2.04 ± 0.82	0.026
Range	1–6		1–6	
Employment status				
Not employed	118 (69.4%)	>	44 (19.4%)	0.000
Full-time employment	23 (13.5%)		34 (15.0%)	
Part-time employment	29 (17.1%)	<	148 (65.5)	
Missing			1 (0.4%)	
Satisfaction with the financial situation				
Very satisfying for making a good living	26 (15.3%)		50 (22.0%)	0.000
Satisfying	72 (42.4%)	<	153 (67.4%)	
Less satisfying	63 (37.1)	>	22 (9.7%)	
Not satisfying at all	8 (4.7%)		1 (0.4%)	
Missing	1 (0.6%)		1 (0.4%)	

*P* < 0.05 indicates a significant effect at the 0.05 level. <, > shows significant differences and the direction.

**Table 2:** Differences in four dimensions of quality of life and depressive symptoms in Japanese and Austrian mothers.

	Japan (*N* = 170)	Austria (*N* = 227)	*P*	Eta²
Physical quality of life				
M ± SD	63.36 ± 14.13	80.28 ± 12.66	0.000	0.284
Psychological quality of life				
M ± SD	59.14 ± 13.76	73.75 ± 14.50	0.000	0.207
Social quality of life				
M ± SD	63.66 ± 14.70	73.0 ± 19.13	0.000	0.066
Environmental quality of life				
M ± SD	59.96 ± 13.18	80.35 ± 13.17	0.000	0.371

Depression				
M ± SD	1.02 ± 1.20	1.19 ± 1.09	0.129	0.006

*P* < 0.05 indicates a significant effect at the 0.05 level.

Eta² = effect size, showing the strength of the study.

**Table 3:** Differences in stress level, sense of coherence, importance of a child, difficulty of child rearing, gender roles, and support of mothers in Japanese and Austrian mothers.

	Japan *N* = 170	Austria *N* = 227	*P*	Eta²
Stress level				
M ± SD	47.75 ± 25.35	49.34 ± 24.19	0.528	0.001

Sense of coherence				
M ± SD	57.82 ± 12.17	68.67 ± 12.20	0.000	0.164

Gender orientation				
General role^*^				
M ± SD	3.68 ± 1.15	4.22 ± 0.86	0.000	0.068
“Men and women should share their duties at home and at their jobs”				
Mothers role^*^				
M ± SD	2.56 ± 1.21	2.88 ± 1.02	0.005	0.021
“It is the mother's responsibility to care for the children”				
Fathers role^*^				
M ± SD	4.05 ± 0.97	4.08 ± 0.90	0.747	0.000
“A father should spend much time with the children during the week, not only at weekends”				
Women's role^*^				
M ± SD	2.55 ± 1.03	2.06 ± 0.976	0.000	0.056
“A woman should mainly care for her family not for her job”				
Men's role^*^				
M ± SD	3.07 ± 1.18	2.54 ± 1.14	0.000	0.048
“A man should be the main financial resource for a family”				

Importance of a child^***^				
M ± SD	6.17 ± 1.19	6.37 ± 1.16	0.090	0.007
Difficulty of childrearing^***^				
M ± SD	4.05 ± 1.77	3.24 ± 1.84	0.000	0.047

Social support				
Social support of important others^**^				
M ± SD	23.45 ± 4.80	25.90 ± 4.35	0.000	0.067
Social support of the family^**^				
M ± SD	23.16 ± 5.00	24.00 ± 5.51	0.119	0.006
Social support of friends^**^				
M ± SD	20.63 ± 5.62	24.04 ± 5.23	0.000	0.090
From the partner^***^				
M ± SD	5.51 ± 1.68	5.94 ± 1.69	0.013	0.016

^*^Answers range from 1 (do not agree) to 5 (agree completely).

^**^Answers are the sum of 4 questions ranging from 1 (very strongly disagree) to 5 (very strongly agree). The total range is between 4 and 28.

^***^Answers range from 1 (very strongly disagree) to 7 (very strongly agree).

*P* < 0.05 indicates a significant effect at the 0.05 level.

Eta² = effect size, showing the strength of the study.

**Table 4:** Demographic and psychological impact factors on depression and quality of life.

Explanatory variables	Criterion variables									
	Depression		Physical QOL		Psychological QOL		Social QOL		Environmental QOL	
	Austrian	Japanese	Austrian	Japanese	Austrian	Japanese	Austrian	Japanese	Austrian	Japanese
Age				−0.144						
Number of children										

Importance of a child										
Difficulty of childrearing										

Satisfaction with income			0.117		0.213				0.397	0.294

Stress	0.154	0.244	−0.217	−0.262		−0.211				

Sense of coherence	−0.582	−0.333	0.373	0.258	0.599	0.429	0.242	0.255	0.283	0.253

Social support of family		−0.166		0.289	0.120	0.310	0.226			
Social support of friends							0.282	0.403	0.182	
Social support of important others										0.275

Support from partner							0.254	0.204		

Mother's role								−0.197		
Father's role								−0.164		

Women's role									−0.120	
Men's role										

General role										

Adjusted *R*-square	0.422	0.342	0.272	0.423	0.529	0.577	0.427	0.506	0.374	0.347
*F*	80.19	29.22	27.99	30.93	82.29	74.58	41.18	34.45	33.43	29.90
df (reg), df (err)	2, 215	3, 160	3, 214	4, 159	3, 214	3, 159	4, 212	5, 158	4, 213	3, 160
